# 2,3-Diamino­pyridinium 4-nitro­benzoate

**DOI:** 10.1107/S160053680902100X

**Published:** 2009-06-06

**Authors:** Kasthuri Balasubramani, Hoong-Kun Fun

**Affiliations:** a X-ray Crystallography Unit, School of Physics, Universiti Sains Malaysia, 11800 USM, Penang, Malaysia

## Abstract

In the title salt, C_5_H_8_N_3_^+^·C_7_H_4_NO_4_^−^, the pyridine N atom of the 2,3-diamino­pyridine mol­ecule is protonated. The protonated N atom and one of the two 2-amino groups are hydrogen bonded to the 4-nitro­benzoate anion through a pair of N—H⋯O hydrogen bonds, forming an *R*_2_^2^(8) ring motif. The carboxyl­ate mean plane of the 4-nitro­benzoate anion is twisted by 3.77 (5)° from the attached ring and the nitro group is similarly twisted by 2.28 (10)°. In the crystal, the mol­ecules are linked by N—H⋯O and C—H⋯O inter­actions into sheets parallel to (100).

## Related literature

For substituted pyridines, see: Pozharski *et al.* (1997 ▶); Katritzky *et al.* (1996 ▶); Jeffrey & Saenger (1991 ▶); Jeffrey (1997 ▶); Scheiner (1997 ▶). For hydrogen-bond motifs, see: Bernstein *et al.* (1995 ▶). For the stability of the temperature controller used in the data collection, see: Cosier & Glazer (1986 ▶).
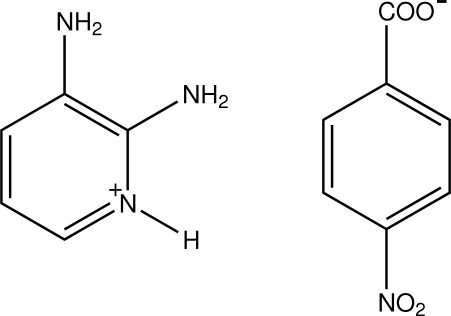


## Experimental

### 

#### Crystal data

C_5_H_8_N_3_^+^·C_7_H_4_NO_4_^−^
*M* *_r_* = 276.26
Monoclinic, 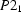

*a* = 8.0827 (2) Å
*b* = 6.7365 (1) Å
*c* = 11.4489 (3) Å
β = 101.967 (1)°
*V* = 609.83 (2) Å^3^
*Z* = 2
Mo *K*α radiation
μ = 0.12 mm^−1^
*T* = 100 K
0.25 × 0.17 × 0.10 mm

#### Data collection

Bruker SMART APEXII CCD area-detector diffractometer
Absorption correction: multi-scan (*SADABS*; Bruker, 2005 ▶) *T*_min_ = 0.972, *T*_max_ = 0.988
11659 measured reflections
2808 independent reflections
2155 reflections with *I* > 2σ(*I*)
*R*_int_ = 0.045

#### Refinement

*R*[*F* ^2^ > 2σ(*F* ^2^)] = 0.052
*wR*(*F* ^2^) = 0.116
*S* = 1.04
2808 reflections
229 parameters
1 restraint
H atoms treated by a mixture of independent and constrained refinement
Δρ_max_ = 0.43 e Å^−3^
Δρ_min_ = −0.30 e Å^−3^

### 

Data collection: *APEX2* (Bruker, 2005 ▶); cell refinement: *SAINT* (Bruker, 2005 ▶); data reduction: *SAINT*; program(s) used to solve structure: *SHELXTL* (Sheldrick, 2008 ▶); program(s) used to refine structure: *SHELXTL*; molecular graphics: *SHELXTL*software used to prepare material for publication: *SHELXTL* and *PLATON* (Spek, 2009 ▶).

## Supplementary Material

Crystal structure: contains datablocks global, I. DOI: 10.1107/S160053680902100X/tk2462sup1.cif

Structure factors: contains datablocks I. DOI: 10.1107/S160053680902100X/tk2462Isup2.hkl

Additional supplementary materials:  crystallographic information; 3D view; checkCIF report

## Figures and Tables

**Table 1:** Hydrogen-bond geometry (Å, °)

*D*—H⋯*A*	*D*—H	H⋯*A*	*D*⋯*A*	*D*—H⋯*A*
N1—H1*N*1⋯O4	0.99 (3)	1.70 (3)	2.671 (2)	167 (3)
N2—H1*N*2⋯O3	0.89 (3)	2.01 (3)	2.901 (2)	178 (5)
N2—H2*N*2⋯O3^i^	0.86 (2)	2.06 (2)	2.903 (2)	171 (2)
N3—H1*N*3⋯O3^i^	0.85 (3)	2.14 (3)	2.951 (3)	159 (2)
N3—H2*N*3⋯O2^ii^	0.82 (3)	2.34 (3)	3.140 (2)	165 (3)
C10—H10*A*⋯O1^ii^	0.98 (3)	2.53 (3)	3.507 (2)	177 (2)
C11—H11*A*⋯O4^iii^	0.99 (2)	2.56 (2)	3.216 (3)	124 (2)

Symmetry codes: (i) 
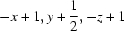
; (ii) 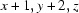; (iii) 
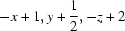
.

**Fig. 1.**
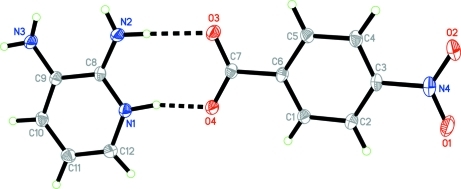
The molecular structures of the ions in (I), illustrating the primary mode of association between them, showing 50% probability displacement ellipsoids and the atom numbering scheme. Dashed lines indicate the hydrogen bonding.

**Fig. 2.**
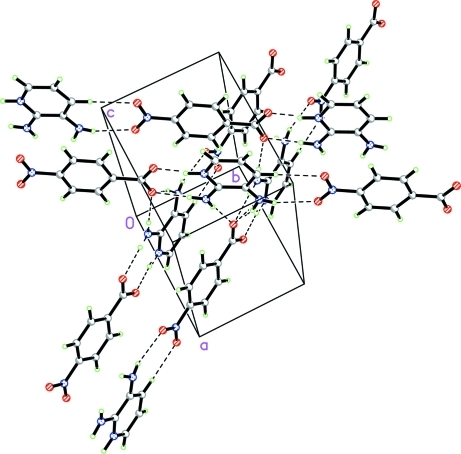
The crystal packing of (I). Dashed lines indicate the hydrogen bondings.

## supplementary crystallographic information

### Comment

Pyridine and its derivatives play an important role in heterocyclic chemistry (Pozharski *et al.*, 1997; Katritzky *et al.*, 1996). Pyridine and its substituted derivatives are often involved in hydrogen bonding interactions (Jeffrey & Saenger, 1991; Jeffrey, 1997; Scheiner, 1997). In order to study some interesting hydrogen bonding interactions, the synthesis and structure of the title salt (I) is presented here.

The asymmetric unit of (I), Fig. 1, contains a protonated 2,3-diaminopyridinium cation and a 4-nitrobenzoate anion. In the 2,3-diaminopyridinium cation, a wide angle (123.62 (17)°) is subtended at the protonated N1 atom. The 2,3-diaminopyridinium cation is planar, with a maximum deviation of 0.005 (2)Å for atom C1. The carboxylate group is twisted slightly from the ring; the dihedral angle between C1—C6 and O3/O4/C7/C6 planes is 5.41 (10)°. The nitro group is also slightly twisted away from its attached benzene ring by 2.28 (10)°.

In the crystal packing, Fig. 2, the protonated N1 atom and the 2-amino group (N2) is hydrogen-bonded to the carboxylate oxygen atoms (O3 and O4) *via* a pair of N—H···O hydrogen bonds forming a ring motif, *R*_2_^2^(8) (Bernstein *et al.*, 1995). The 2-amino groups (N2 and N3) are involved in N—H···O3 hydrogen bonding interactions to form a *R*^1^_2_(7) ring motif. One of the amino group hydrogen atoms, H2N3, and the ring hydrogen atom, H10A, are connected to the 4-nitro group oxygen atoms (O1 and O2) to form an *R*_2_^2^(8) ring motif (Table 1 and Fig. 2). These molecules are linked by these interactions into sheets parallel to (100). The crystal structure is further stabilized by a π-π stacking interactions between the aminopyridine- and carboxylate-rings with centroid-to-centroid distances of 3.8343 (10) Å.

### Experimental

Hot methanol solutions (20 ml) of 2,3-diaminopyridine (27 mg, Aldrich) and 4-nitrobenzoic acid (42 mg, Merck) were mixed and warmed over a heating magnetic stirrer for 5 minutes. The resulting solution was allowed to cool slowly at room temperature. Crystals of (I) appeared from the mother liquor after a few days.

### Refinement

All the H atoms were located from the difference Fourier map [N–H = 0.82 (3)–0.99 (3)Å and C–H = 0.91 (2)–0.99 (2) Å] and allowed to refine freely. In the absence of significant anomalous scattering effects, 2144 Friedel pairs were merged.

### Crystal data

**Table:**

C_5_H_8_N_3_^+^·C_7_H_4_NO_4_^−^	*F*(000) = 288
*M**_r_* = 276.26	*D*_x_ = 1.504 Mg m^−^^3^
Monoclinic, *P*2_1_	Mo *K*α radiation, λ = 0.71073 Å
Hall symbol: P 2yb	Cell parameters from 2526 reflections
*a* = 8.0827 (2) Å	θ = 2.8–31.7°
*b* = 6.7365 (1) Å	µ = 0.12 mm^−^^1^
*c* = 11.4489 (3) Å	*T* = 100 K
β = 101.967 (1)°	Block, brown
*V* = 609.83 (2) Å^3^	0.25 × 0.17 × 0.10 mm
*Z* = 2	

### Data collection

**Table:**

Bruker SMART APEXII CCD area-detector diffractometer	2808 independent reflections
Radiation source: fine-focus sealed tube	2155 reflections with *I* > 2σ(*I*)
graphite	*R*_int_ = 0.045
φ and ω scans	θ_max_ = 34.9°, θ_min_ = 2.6°
Absorption correction: multi-scan (*SADABS*; Bruker, 2005)	*h* = −12→12
*T*_min_ = 0.972, *T*_max_ = 0.988	*k* = −10→10
11659 measured reflections	*l* = −17→18

### Refinement

**Table:**

Refinement on *F*^2^	Primary atom site location: structure-invariant direct methods
Least-squares matrix: full	Secondary atom site location: difference Fourier map
*R*[*F*^2^ > 2σ(*F*^2^)] = 0.052	Hydrogen site location: inferred from neighbouring sites
*wR*(*F*^2^) = 0.116	H atoms treated by a mixture of independent and constrained refinement
*S* = 1.04	*w* = 1/[σ^2^(*F*_o_^2^) + (0.0571*P*)^2^ + 0.026*P*] where *P* = (*F*_o_^2^ + 2*F*_c_^2^)/3
2808 reflections	(Δ/σ)_max_ < 0.001
229 parameters	Δρ_max_ = 0.43 e Å^−^^3^
1 restraint	Δρ_min_ = −0.30 e Å^−^^3^

### Special details

**Table:**

Experimental. The crystal was placed in the cold stream of an Oxford Cyrosystems Cobra open-flow nitrogen cryostat (Cosier & Glazer, 1986) operating at 100.0 (1) K.
Geometry. All s.u.'s (except the s.u. in the dihedral angle between two l.s. planes) are estimated using the full covariance matrix. The cell s.u.'s are taken into account individually in the estimation of s.u.'s in distances, angles and torsion angles; correlations between s.u.'s in cell parameters are only used when they are defined by crystal symmetry. An approximate (isotropic) treatment of cell s.u.'s is used for estimating s.u.'s involving l.s. planes.
Refinement. Refinement of F^2^ against ALL reflections. The weighted R-factor wR and goodness of fit S are based on F^2^, conventional R-factors R are based on F, with F set to zero for negative F^2^. The threshold expression of F^2^ > σ(F^2^) is used only for calculating R-factors(gt) etc. and is not relevant to the choice of reflections for refinement. R-factors based on F^2^ are statistically about twice as large as those based on F, and R- factors based on ALL data will be even larger.

### Fractional atomic coordinates and isotropic or equivalent isotropic displacement parameters (Å^2^)

**Table:**

	*x*	*y*	*z*	*U*_iso_*/*U*_eq_	
O1	−0.0506 (2)	−0.7085 (2)	0.85306 (17)	0.0306 (4)	
O2	−0.04637 (19)	−0.7831 (2)	0.66897 (16)	0.0269 (4)	
O3	0.35100 (19)	0.1109 (2)	0.60686 (14)	0.0223 (3)	
O4	0.35695 (19)	0.1713 (2)	0.79972 (14)	0.0210 (3)	
N4	−0.0141 (2)	−0.6717 (3)	0.75606 (18)	0.0213 (4)	
C1	0.1816 (2)	−0.1740 (3)	0.82546 (19)	0.0178 (4)	
C2	0.1021 (2)	−0.3532 (3)	0.8391 (2)	0.0183 (4)	
C3	0.0723 (2)	−0.4824 (3)	0.7432 (2)	0.0178 (4)	
C4	0.1172 (2)	−0.4421 (3)	0.6355 (2)	0.0196 (4)	
C5	0.1973 (2)	−0.2624 (3)	0.6234 (2)	0.0180 (4)	
C6	0.2309 (2)	−0.1287 (3)	0.71862 (19)	0.0162 (4)	
C7	0.3201 (2)	0.0666 (3)	0.70675 (18)	0.0169 (4)	
N1	0.5459 (2)	0.4983 (2)	0.80784 (16)	0.0179 (3)	
N2	0.5600 (2)	0.4619 (3)	0.60992 (18)	0.0213 (4)	
N3	0.7324 (2)	0.8308 (3)	0.62077 (18)	0.0212 (4)	
C8	0.5959 (2)	0.5681 (3)	0.71027 (19)	0.0166 (4)	
C9	0.6873 (2)	0.7522 (3)	0.72040 (19)	0.0168 (4)	
C10	0.7217 (2)	0.8451 (3)	0.8301 (2)	0.0197 (4)	
C11	0.6717 (2)	0.7629 (3)	0.9295 (2)	0.0213 (4)	
C12	0.5820 (2)	0.5895 (3)	0.91644 (19)	0.0199 (4)	
H1A	0.201 (3)	−0.084 (4)	0.887 (2)	0.020 (6)*	
H2A	0.068 (3)	−0.384 (4)	0.914 (2)	0.020 (6)*	
H4A	0.089 (3)	−0.535 (5)	0.569 (2)	0.033 (7)*	
H5A	0.232 (3)	−0.233 (4)	0.547 (2)	0.021 (6)*	
H10A	0.782 (3)	0.972 (5)	0.834 (2)	0.029 (7)*	
H11A	0.711 (3)	0.821 (4)	1.010 (2)	0.027 (7)*	
H12A	0.540 (3)	0.527 (4)	0.978 (2)	0.021 (6)*	
H1N1	0.478 (3)	0.375 (5)	0.793 (3)	0.038 (8)*	
H1N2	0.497 (4)	0.353 (5)	0.608 (3)	0.038 (8)*	
H2N2	0.579 (3)	0.496 (4)	0.542 (2)	0.017 (6)*	
H1N3	0.735 (3)	0.759 (5)	0.560 (2)	0.025 (7)*	
H2N3	0.806 (4)	0.917 (5)	0.635 (3)	0.037 (8)*	

### Atomic displacement parameters (Å^2^)

**Table:**

	*U*^11^	*U*^22^	*U*^33^	*U*^12^	*U*^13^	*U*^23^
O1	0.0374 (9)	0.0213 (8)	0.0368 (10)	−0.0086 (6)	0.0162 (8)	0.0038 (7)
O2	0.0274 (7)	0.0170 (7)	0.0348 (10)	−0.0058 (6)	0.0027 (7)	−0.0027 (7)
O3	0.0293 (7)	0.0175 (6)	0.0226 (8)	−0.0035 (5)	0.0109 (6)	0.0002 (6)
O4	0.0281 (7)	0.0164 (6)	0.0199 (8)	−0.0056 (5)	0.0083 (6)	−0.0023 (6)
N4	0.0165 (7)	0.0146 (7)	0.0320 (10)	−0.0015 (6)	0.0030 (7)	0.0026 (8)
C1	0.0213 (8)	0.0139 (8)	0.0187 (10)	−0.0012 (7)	0.0053 (7)	−0.0008 (8)
C2	0.0185 (8)	0.0155 (8)	0.0215 (10)	−0.0007 (6)	0.0054 (7)	0.0030 (8)
C3	0.0166 (8)	0.0116 (7)	0.0255 (11)	−0.0015 (6)	0.0053 (7)	0.0010 (7)
C4	0.0216 (8)	0.0132 (8)	0.0249 (11)	−0.0008 (6)	0.0067 (8)	−0.0015 (8)
C5	0.0205 (8)	0.0146 (8)	0.0196 (10)	−0.0010 (7)	0.0056 (7)	−0.0018 (8)
C6	0.0176 (8)	0.0111 (8)	0.0201 (10)	0.0012 (6)	0.0043 (7)	0.0032 (7)
C7	0.0180 (8)	0.0128 (8)	0.0208 (10)	0.0003 (6)	0.0058 (7)	0.0008 (8)
N1	0.0188 (7)	0.0161 (7)	0.0191 (9)	−0.0021 (6)	0.0048 (6)	0.0004 (7)
N2	0.0300 (9)	0.0176 (8)	0.0182 (9)	−0.0056 (6)	0.0094 (7)	−0.0014 (7)
N3	0.0273 (8)	0.0178 (7)	0.0201 (9)	−0.0061 (7)	0.0089 (7)	−0.0003 (8)
C8	0.0158 (7)	0.0136 (8)	0.0208 (10)	−0.0012 (6)	0.0046 (7)	0.0007 (8)
C9	0.0157 (7)	0.0161 (8)	0.0189 (9)	0.0003 (6)	0.0042 (6)	0.0023 (8)
C10	0.0199 (8)	0.0168 (8)	0.0228 (11)	−0.0021 (7)	0.0055 (7)	−0.0018 (8)
C11	0.0210 (8)	0.0225 (9)	0.0206 (11)	−0.0001 (7)	0.0051 (7)	−0.0028 (8)
C12	0.0201 (8)	0.0223 (9)	0.0176 (10)	−0.0010 (7)	0.0045 (7)	0.0004 (8)

### Geometric parameters (Å, °)

**Table:**

O1—N4	1.232 (2)	N1—C8	1.349 (3)
O2—N4	1.232 (2)	N1—C12	1.363 (3)
O3—C7	1.256 (2)	N1—H1N1	0.99 (3)
O4—C7	1.260 (2)	N2—C8	1.333 (3)
N4—C3	1.476 (2)	N2—H1N2	0.89 (3)
C1—C2	1.391 (3)	N2—H2N2	0.85 (3)
C1—C6	1.397 (3)	N3—C9	1.373 (3)
C1—H1A	0.92 (3)	N3—H1N3	0.85 (3)
C2—C3	1.383 (3)	N3—H2N3	0.82 (3)
C2—H2A	0.98 (3)	C8—C9	1.435 (3)
C3—C4	1.382 (3)	C9—C10	1.379 (3)
C4—C5	1.394 (3)	C10—C11	1.399 (3)
C4—H4A	0.98 (3)	C10—H10A	0.98 (3)
C5—C6	1.396 (3)	C11—C12	1.367 (3)
C5—H5A	0.99 (3)	C11—H11A	0.99 (3)
C6—C7	1.520 (3)	C12—H12A	0.94 (3)
			
O2—N4—O1	123.84 (17)	C8—N1—C12	123.62 (17)
O2—N4—C3	118.06 (18)	C8—N1—H1N1	113.8 (17)
O1—N4—C3	118.10 (18)	C12—N1—H1N1	122.6 (17)
C2—C1—C6	120.69 (19)	C8—N2—H1N2	119.0 (19)
C2—C1—H1A	119.4 (17)	C8—N2—H2N2	126.1 (17)
C6—C1—H1A	119.9 (17)	H1N2—N2—H2N2	114 (2)
C3—C2—C1	117.7 (2)	C9—N3—H1N3	121 (2)
C3—C2—H2A	122.2 (16)	C9—N3—H2N3	114 (2)
C1—C2—H2A	120.1 (16)	H1N3—N3—H2N3	115 (3)
C4—C3—C2	123.38 (18)	N2—C8—N1	118.49 (17)
C4—C3—N4	118.44 (18)	N2—C8—C9	123.34 (19)
C2—C3—N4	118.18 (18)	N1—C8—C9	118.16 (18)
C3—C4—C5	118.21 (19)	N3—C9—C10	122.90 (18)
C3—C4—H4A	120.7 (17)	N3—C9—C8	119.10 (19)
C5—C4—H4A	121.1 (17)	C10—C9—C8	117.97 (18)
C4—C5—C6	120.13 (19)	C9—C10—C11	121.58 (18)
C4—C5—H5A	118.6 (16)	C9—C10—H10A	116.5 (16)
C6—C5—H5A	121.2 (16)	C11—C10—H10A	121.9 (16)
C5—C6—C1	119.87 (17)	C12—C11—C10	119.1 (2)
C5—C6—C7	120.51 (17)	C12—C11—H11A	120.1 (16)
C1—C6—C7	119.62 (17)	C10—C11—H11A	120.5 (16)
O3—C7—O4	125.37 (18)	N1—C12—C11	119.6 (2)
O3—C7—C6	118.35 (18)	N1—C12—H12A	115.8 (16)
O4—C7—C6	116.28 (17)	C11—C12—H12A	124.6 (16)
			
C6—C1—C2—C3	−0.8 (3)	C1—C6—C7—O3	−174.40 (17)
C1—C2—C3—C4	−0.1 (3)	C5—C6—C7—O4	−174.72 (17)
C1—C2—C3—N4	−179.16 (16)	C1—C6—C7—O4	5.6 (2)
O2—N4—C3—C4	−1.9 (3)	C12—N1—C8—N2	177.00 (18)
O1—N4—C3—C4	178.55 (18)	C12—N1—C8—C9	−2.2 (3)
O2—N4—C3—C2	177.21 (18)	N2—C8—C9—N3	3.9 (3)
O1—N4—C3—C2	−2.3 (3)	N1—C8—C9—N3	−176.90 (17)
C2—C3—C4—C5	0.4 (3)	N2—C8—C9—C10	−177.88 (18)
N4—C3—C4—C5	179.44 (17)	N1—C8—C9—C10	1.3 (2)
C3—C4—C5—C6	0.2 (3)	N3—C9—C10—C11	178.94 (19)
C4—C5—C6—C1	−1.0 (3)	C8—C9—C10—C11	0.8 (3)
C4—C5—C6—C7	179.32 (16)	C9—C10—C11—C12	−2.1 (3)
C2—C1—C6—C5	1.3 (3)	C8—N1—C12—C11	0.9 (3)
C2—C1—C6—C7	−179.02 (17)	C10—C11—C12—N1	1.3 (3)
C5—C6—C7—O3	5.3 (3)		

### Hydrogen-bond geometry (Å, °)

**Table:**

*D*—H···*A*	*D*—H	H···*A*	*D*···*A*	*D*—H···*A*
N1—H1N1···O4	0.99 (3)	1.70 (3)	2.671 (2)	167 (3)
N2—H1N2···O3	0.89 (3)	2.01 (3)	2.901 (2)	178 (5)
N2—H2N2···O3^i^	0.86 (2)	2.06 (2)	2.903 (2)	171 (2)
N3—H1N3···O3^i^	0.85 (3)	2.14 (3)	2.951 (3)	159 (2)
N3—H2N3···O2^ii^	0.82 (3)	2.34 (3)	3.140 (2)	165 (3)
C10—H10A···O1^ii^	0.98 (3)	2.53 (3)	3.507 (2)	176.9 (16)
C11—H11A···O4^iii^	0.99 (2)	2.56 (2)	3.216 (3)	123.5 (19)

Symmetry codes: (i) −*x*+1, *y*+1/2, −*z*+1; (ii) *x*+1, *y*+2, *z*; (iii) −*x*+1, *y*+1/2, −*z*+2.
